# *In Vitro* and Sensory Evaluation of Capsaicin-Loaded Nanoformulations

**DOI:** 10.1371/journal.pone.0141017

**Published:** 2015-10-22

**Authors:** Mathias Kaiser, Benedikt Kirsch, Hannah Hauser, Désirée Schneider, Ingrid Seuß-Baum, Francisco M. Goycoolea

**Affiliations:** 1 Institute of Plant Biology and Biotechnology (IBBP), Westfälische Wilhelms-Universität Münster, Münster, North Rhine-Westphalia, Germany; 2 Department of Food Technology, University of Applied Sciences Fulda, Fulda, Hesse, Germany; German Institute of Human Nutrition Potsdam-Rehbruecke, GERMANY

## Abstract

Capsaicin has known health beneficial and therapeutic properties. It is also able to enhance the permeability of drugs across epithelial tissues. Unfortunately, due to its pungency the oral administration of capsaicin is limited. To this end, we assessed the effect of nanoencapsulation of capsaicin, under the hypothesis that this would reduce its pungency. Core-shell nanocapsules with an oily core and stabilized with phospholipids were used. This system was used with or without chitosan coating. In this work, we investigated the in vitro release behavior of capsaicin-loaded formulations in different physiological media (including simulated saliva fluid). We also evaluated the influence of encapsulation of capsaicin on the cell viability of buccal cells (TR146). To study the changes in pungency after encapsulation we carried out a sensory analysis with a trained panel of 24 students. The *in vitro* release study showed that the systems discharged capsaicin slowly in a monotonic manner and that the chitosan coating had an effect on the release profile. The cytotoxic response of TR146 cells to capsaicin at a concentration of 500 μM, which was evident for the free compound, was reduced following its encapsulation. The sensory study revealed that a chitosan coating results in a lower threshold of perception of the formulation. The nanoencapsulation of capsaicin resulted in attenuation of the sensation of pungency significantly. However, the presence of a chitosan shell around the nanoformulations did not mask the pungency, when compared with uncoated systems.

## Introduction

Capsaicin (N-(4-hydroxy-3-methoxybenzyl)-8-methyl-trans-6nonenamide) is a pungent alkaloid found in plants of the genus Capsicum (for example chili peppers) in concentrations between 0.3–0.5% [1]. Capsaicin is an agonist to TRPV1 receptors causing burn and pain sensations on skin and mucus membranes [2]. Taken orally, it influences the cardiovascular system, induces an increase of salivation and gastric secretion and gastrointestinal disorders in higher dosages [3,4]. Furthermore, capsaicin is also known for its antioxidant, anti-inflammatory, anticarcinogenic and antifungal effects [5,6]. In addition, it is also known as promoter of energy metabolism, suppresses fat accumulation [7] and is able to reversibly open cellular tight junctions [8]. However, due to its strong pungency, its toxicity at high doses [9] and low water solubility [10], the therapeutic administration of capsaicin is limited. In previous studies, we have evaluated the effect of associating capsaicin in colloidal nanosystems on the phyisical and in vitro biopharmaceutical properties, particularly on the cell permeability [11,12]. In such studies we demonstrated that the encapsulation of capsaicin has a cytoprotective effect and that the cellular permeability of a model co-administered macromolecular substance (FITC-dextran) can be enhanced. Thus, capsaicin-loaded nanocapsules can be conceived as an innovative drug delivery platform. In this study two formulations have been investigated: The first consists of a lipophilic core comprised by an inert mixture of fatty acids which is used for pharmaceutical formulations (Miglyol) which is stabilized by a layer of lecithin. The second formulation is identical to the first one but it is additionally coated by a layer of chitosan, a natural aminopolysaccharide comprising glucosamine and N-acetyl glucosamine units. This polymer is known to have beneficial properties like a mucoadhesive effect [13] and the ability to interfere with cellular tight junctions [14]. Capsaicin was loaded into the oily core of both systems. By means of these formulations a co-administered substance could be transported to areas of the body protected by biological barriers [12].

Several nano- and microparticulate systems containing capsaicin have been developed in recent years [11,15–21]. Most of these formulations are based on natural polymers and have the purpose to make capsaicin compatible to the aqueous environment in organisms due to its low water solubility [10]. The use of capsaicin loaded drug delivery systems to improve the bioavailability of other drugs has not been extensively studied so far.

In the present study we investigated the *in vitro* release behavior of the formulations in simulated saliva fluid, evaluated the cytotoxicity of free and encapsulated capsaicin to buccal cells (TR146) and carried out a sensory analysis to study the effect of encapsulation on sensation of pungency to evaluate their feasibility for oral administration. We found that the nanoformulations released capsaicin under different profiles in physiological media, attenuated the cytotoxic response of buccal cells and reduced the pungency sensation. While the chitosan coating had an effect on the *in vitro* release profiles, it was not evident from the sensory analysis.

## Materials and Methods

### Chitosan

Ultrapure biomedical grade chitosan (Heppe 70/5, Batch No. 212-140311-02) was purchased from HMC^+^ GmbH (Halle/Saale, Germany). By measuring its intrinsic viscosity in 0.3 M acetic acid/0.2 M sodium acetate at 25°C [22] the molecular weight of 17,600 Da was determined and the degree of acetylation of 32.4% was determined by ^1^H NMR spectroscopy. Ultrapure MilliQ water was used throughout.

### Preparation of the nanoformulations

The chitosan-coated nanocapsules were prepared as previously described with modifications [11,12]. Briefly, for the *in vitro* experiments 400 μl of a 100 mg/ml ethanolic lecithin solution (Epikuron 145 V, Cargill texturing solutions Deutschland GmbH &Co. KG, Hamburg, Germany) was mixed with 530 μl of the capsaicin stock solution (24 mg/ml). This was supplemented with 125 μl Miglyol 812 N (Sasol GmbH, Witten, Germany) and 9.5 ml ethanol. The organic solution was immediately poured into 20 ml chitosan in the aqueous phase (0.5 mg/ml in 5% stoichiometric excess of HCl). The milky mixture was concentrated in a rotavapor (Büchi R-210, Büchi Labortechnik GmbH, Essen, Germany) at 50°C until 3.5–4.0 ml remained and the volume was topped up to 4.0 ml with milliQ water if necessary to yield a final capsaicin concentration of ~10 mM. The nanoemulsions were prepared using the same procedure but without including chitosan. Unloaded nanocapsules and nanoemulsions were prepared by replacing the capsaicin solution with ethanol. For the sensory studies larger batches (25-fold greater than the above described amounts) with a final volume of 500 ml were produced. In this case the loaded formulations were adjusted to a capsaicin concentration of 0.8 ppm (2.6 μM) or 8 ppm (26 μM).

### Simulated saliva fluid

Simulated saliva fluid was prepared by adapting a previously reported formulation [23]. To 1 l of milliQ water 2.38 g Na_2_HPO_4_, 0.19 g KH_2_PO_4_ and 8.00 g NaCl were added. This solution was adjusted with phosphoric acid to pH 6.75. For the stability measurements, to this formulation, α-amylase (α-Amylase from human saliva, Type XIII-A, lyophilized powder, 300–1.500 units/mg protein, Sigma-Aldrich, Steinheim, Germany) was added. An amount of 13 U was added which resembles the conditions of resting saliva of healthy adults [24].

### Size and zeta potential

The size distribution of the nanoformulations was determined by dynamic light scattering with non-invasive back scattering (DLS-NIBS) at a measurement angle of 173°. The zeta potential was measured by mixed laser Doppler velocimetry and phase analysis light scattering (M3–PALS). A Malvern Zetasizer NanoZS (Malvern Instruments Ltd., Worcestershire, UK) equipped with a red laser light (λ = 632.8 nm) was used for both methods. The samples were diluted 1:50 in water for size measurements and 1:50 in 1 mM KCl for zeta potential measurements. The same ratio has been used for the stability measurements in the respective buffers.

### Analysis of capsaicin by HPLC-UV/VIS

HPLC-UV/VIS analysis was carried out using a Jasco HPLC system (Jasco GmbH, Gross-Umstadt, Germany) comprising a three-line degasser (DG-2080-53), a ternary gradient unit (LG-2080-02S), a semi-micro HPLC pump (PU-2085Plus), an autosampler (X-LC™ 3159AS), an intelligent column thermostat (CO-2060 Plus) equipped with a Kinetex C-18 reversed phase column (2.6 μm; Aeris widepore 3.6u XB-C; 150 x 2.1 mm; void volume 264 μL; S/N: 698087–3; Phenomenex, Torrance, USA) thermostated at 40°C and a UV/Vis detector (X-L™ 3075UV). Samples were eluted using the gradient shown in Table 1 at a flow rate of 0.6 ml/min. Capsaicin was detected at λ = 228 nm at a retention time of 1.06 minutes after injection. The total runtime of the method was 3 min.

**Table 1:** HPLC gradient profile for the detection of capsaicin.

Time (min)	H_2_O (%)	Acetonitrile (%)
0	50	50
1.5	25	75
1.7	25	75
2	50	50
3	50	50

### *In vitro* capsaicin release assay

An 800-μl aliquot of each formulation was transferred to a dialysis tube (Pure-a-lyzer Maxi 0.1–3.0 ml, Mw cut-off = 6000 Da, Sigma-Aldrich GmbH, Steinheim, Germany) and placed in a glass beaker containing 79.2 ml of release medium previously equilibrated at 37°C in an incubator. Every hour, a 500-μl aliquot of medium was removed and replaced with the same volume of buffer. The capsaicin content of the aliquots was determined by HPLC-UV/VIS as described above. The transport of capsaicin from the nanoformulations into the medium was analyzed by fitting the data to the empirical equation known as Korsmeyer-Peppas drug release model [25]. This model is commonly used to characterize the release profiles of drugs in delivery formulations [26]:

$$\frac{M_{t}}{M_{\infty }}=\;{kt}^{n}$$ (1)

where M_t_ is the mass of capsaicin released at time t. The parameter M_∞_ represents the total mass of capsaicin to be released and k is a constant that depends on the structural characteristics of the nanoformulation and the solvent/material interactions. The exponent *n* is used to indicate the type of diffusion. When n = 0.43 the diffusion is Fickian, when n = 0.85 diffusion involves Case II transport, when n is between these values it is anomalous, and when n > 0.85 diffusion involves super-Case II transport and reflects the craze and fracture of the material due to osmotic pressure differences.

### Cell culture

TR146 cells (Sigma-Aldrich GmbH, Steinheim, Germany) were cultured in 75 cm^2^ flasks using Ham's F12 Nutrient Mixture supplemented with 10% fetal bovine serum, 1% L-glutamine (200 mM) and 1% penicillin-streptomycin (10000 units penicillin, 10000 units of streptomycin in 0.9% NaCl). The cultures were maintained in a humid atmosphere at 37°C with 5% CO_2_ (Sanyo MCO-19AIC, Panasonic Biomedical Sales Europe BV, AZ Etten Leur, Netherlands). Cells from passages 10–17 were used for all experiments, which were carried out as independent triplicates on different days. After reaching microscopic confluence, the cells were washed with 10 ml phosphate buffered saline (PBS) and trypsinized with 10 ml 0.05% trypsin in EDTA (1x) buffer. After detachment, 10 ml of medium was added to the trypsin buffer. The cell suspension was centrifuged at 1000 rpm for 5 min (Rotina 420 R, Hettich GmbH, Tuttlingen, Germany). The excess of medium was removed and the cell pellet was resuspended in 1 ml medium. A 10-μl aliquot of the cell suspension was diluted with 90 μl trypan blue and the number of cells was counted with an improved Neubauer chamber before seeding. The cells were subcultured by splitting at a ratio of 1:10.

### 3-(4,5-dimethylthiazol-2-yl)-2,5-diphenyltetrazolium bromide (MTT) assay

The cytotoxicity of the nanoformulations and components was evaluated using an MTT assay [27]. Briefly, 100 μl of cell suspension was transferred to each well of a 96-well tissue culture plate (~10^4^ cells per well or ~10^5^ cells/ml) and allowed to attach for 24 h. The cells were washed twice with supplement-free medium before the sample was added and the cells were incubated for 3 h. The samples were removed and replaced with 100 μl supplement-free medium. We prepared an MTT solution in PBS with a concentration of 5 mg/ml of thiazolyl blue tetrazolium bromide and added 25 μl to each well. After 4 h, the medium was again removed and the dye was dissolved in DMSO. After orbital shaking at 300 rpm for 15 min, the absorbance was measured at λ = 570 nm in a microplate reader (Safire, Tecan AG, Salzburg, Austria). Relative viability values were calculated by dividing individual viabilities by the mean of the negative control (untreated cells). 4% Triton X-100 in PBS was used as a positive control.

### Sensory studies

#### Sensory panel

A panel of 24 students (13 males, 11 females) from the Department of Food Technology at the University of Applied Sciences Fulda participated in this study. Their ages ranged between 19 and 27 years and were chosen due to their willingness, availability, and also their basic knowledge about sensory methods. Furthermore it was requested that none of the panelists were allergic to crustaceans. Candies and book vouchers were offered as incentives for their voluntary participation. Within the scope of a corresponding course they had basic experience with sensory methods [28] but were not familiar with the assessment of pungency from previous tests. Before starting the sensory training the subjects provided written and verbal consent for the study procedure. Also the protocol of the experimental procedure was approved by the University of Applied Sciences Fulda Ethics Review Board.

#### Test conditions and sample management

The experiments were performed in a test room according to commonly used standards [29]. The samples were stored at 7°C but were pre-conditioned to room temperature before the test. As a reference sample unencapsulated capsaicin was used. Due to hydrophobic solution behavior it was necessary to add an emulsifier to dissolve capsaicin in water. A stock solution of 1000 ppm capsaicin was prepared. For best emulsification effect 120 mg Tween 80 were dissolved into 100 ml decalcified water. From this solution 20 ml were dissolved in 2 l decalcified water to create a 12 ppm polysorbate 80 solution. In 10 ml of the same solution 10 mg of capsaicin were dissolved. For the best solution effect this mixture was alternately shaken manually and heated in an ultrasonic bath at 40°C until the capsaicin was completely dissolved. To ensure that the panelists were not influenced by the presentation order each sample was encoded with a fully random three digit numerical code and did not differ in appearance or odor. These samples were presented to the panelists in a laboratory glass with a standardized filling of 5 ml.

#### Panel training

Each panelist had to be trained in the assessment of pungency before starting the main experiments. For detecting the thresholds there was a training session with a three-alternative forced-choice (3-AFC) test of loaded and unloaded capsules and emulsions. Based on this procedure all 24 volunteers were able to participate on the main threshold experiments. Furthermore, for dealing with the time intensity test each panelist was trained to perform computer-aided tests using FIZZ (Biosystemes, Version 2.46A, France) software in a total of two whole training sessions and every time before starting the main experiments. In the first intensity training session the panelists were instructed to rate the intensity of the sample according to the guidelines of intensity test on a scale from 0 to 10 with 0 “not detectable” and 10 “very intensive”. Each concentration was measured in duplicate to control that the panelist rated given concentrations identically. In addition to that they were not informed before that every two sample had the same concentration. Only the panelists who rated identical concentrations similarly were further selected for the main study. That means 17 panelists out of 24 volunteers were chosen to participate based on the reproducibility of their assessments. At first sweet samples with aqueous sucrose solutions in the concentrations of 3.0, 6.0 and 12.0 g/l were offered to get the panelist used to this method. Afterwards the panelists were asked to rate samples containing capsaicin. The final concentrations of the samples were 0.5, 1.0, 1.5 and 2.0 ppm. At first the panel rated the magnitude of pungency using FIZZ and afterwards the results were discussed among the group. These concentrations were fixed according to our earlier established method [30] as the following ratings on a 10-point scale: slight (0.5 ppm, designated a 2 on a 10-point scale), moderate (1.0 ppm, designated a 4 on a 10-point scale), strong (1.5 ppm, designated a 6 on a 10-point scale) and very strong (more pungent than the 2.0 ppm solution, designated a rating higher than 6 on a 10-point scale).

#### Sensory determination of nanoformulation thresholds

To evaluate the influence of the taste of the encapsulation material a 3-AFC-test (ISO 13301:2002–09) was used to determine the thresholds of unloaded formulations. For this each panelist got three (nanoemulsions) or five (nanocapsules) rows with each row containing three samples, one differing from the other two in ascending concentrations. The samples did not differ in appearance or odor and the panelists had to find out the odd one. The concentrations refer to the recipe according to the methods section. For the unloaded chitosan-coated nanocapsules concentrations of 0.63, 1.25, 2.5, 5, and 10 ppm of the original solution were tested in the panel against decalcified water.

In preliminary tests with unloaded nanoemulsions participants remarked a feeling of furred tongue. That was the reason why for the nanoemulsion threshold determination only three concentrations (10, 100, and 1000 ppm) were given and the number of panel members was expanded to 34 persons.

#### Sensory determination of capsaicin thresholds in nanoformulations

A 3-AFC-test [31] was also performed for the threshold detection of capsaicin encapsulated in nanoformulations und their unloaded counterparts. For this each panelist got four rows with each row containing three samples, one differing from the other two in ascending concentrations of capsaicin (0.018, 0.035, 0.070 and 0.140 ppm). Both capsaicin loaded capsules and emulsions were tested against their unloaded counterparts.

The 3-AFC-test was chosen because the likelihood of only guessing the right sample is about 33.3% which is quite low in comparison to other threshold tests.

#### Sensory determination of intensity

To compare the intensity of the same capsaicin concentration in different carriers (water, nanocapsules and nanoemulsions) the time intensity test [32] was performed computerized via FIZZ software. Each sample was given to the panelist in laboratory glasses with a constant volume of 5 ml. In each session unencapsulated capsaicin, loaded capsules and loaded emulsion were tested at the same concentrations (2 and 6 ppm) in duplicate. Like in the training sessions panelists were instructed to rate the magnitude of pungency (via clicking the left mouse button on a scale from 0 to 10 with 0 (pungency not detectable) and 10 (very pungent). For each sample the panelists had a maximum time of 10 minutes for rating the burning sensation. After that they had one minute left for a short break and neutralization by using mascarpone toast [30] and decalcified water before continuing with the next sample.

### Data analysis

#### Best estimate threshold (BET)

The BET was calculated according to ISO standard [31]. Therefore the last failed concentration and the next higher concentration with a correct response of each single panelist were determined and averaged geometrically. Out of these individual BETs the BET for the whole group was calculated. Either a one- or three-way ANOVA was performed. The standard deviation was calculated out of the standard deviations of the logarithmic individual BETs.

#### Time intensity

The data for analysis of time intensity was collected by FIZZ software. However, in this study only the maximum of pungency intensity (*I*_max_) was used for further evaluation. The data were reviewed by David test for normal distribution and statistical outliers were eliminated by Dixon test. This is followed by a three-way variance analysis using SPSS (IBM Deutschland GmbH, Version 20, Germany) with the variables panelists, type of encapsulation and replicates in a 5% significance level. Post-hoc-test (Tukey-B) was performed to identify the significant difference between concentrations (2 and 6 ppm) and the type of encapsulation (free capsaicin, loaded nanocapsules, loaded nanoemulsion).

#### Cell experiments

Statistical analysis was carried out using Prism v6.0c (GraphPad Software Inc., La Jolla, USA). All experiments were statistically analyzed using non-parametric tests. The Kruskal-Wallis test in combination with Dunn's multiple comparison test was used. All biological experiments were conducted at least in triplicate.

## Results and Discussion

To evaluate the effect of encapsulation on the pungency of capsaicin we investigated the physicochemical properties of two different systems, studied their interaction with cells using *in vitro* experiments and conducted a sensory analysis to study the influence on pungency sensation.

### Physicochemical properties of nanosystems

The physical characteristics of the nanoformulations used in this study are shown in Table 2. All the systems had a diameter between ~ 115 and ~ 199 nm and the PDI (polydispersity index) ranged between ~ 0.06 and ~ 0.16. The nanoemulsion had a negative zeta potential (~ -35 or ~ -50 mV) while it was positive for the chitosan coated nanoformulations (~ +35 mV). The determined properties of the nanosystems (size, zeta potential, PDI) were in close agreement with previous studies [11,12]. The characteristics of the formulations remained similar even when we did not use acetone in the organic phase during the preparation process as previously reported [33].

**Table 2:** Physicochemical properties of chitosan-coated nanocapsules and nanoemulsions with or without capsaicin.

Nanoformulation	Size (d., nm)	PDI	Zeta potential (mV)
Unloaded NE	133± 6	0.15 ± 0.03	-35.6 ± 6.2
Loaded NE (26 μM)	128 ± 15	0.15 ± 0.02	-34.8 ± 4.7
Loaded NE (10 mM)	140 ± 16	0.16 ± 0.03	-51.1 ± 5.1
Unloaded NC	115 ± 3	0.12 ± 0.01	+33.7 ± 2.8
Loaded NC (26 μM)	121 ± 5	0.13 ± 0.02	+36.4 ± 2.1
Loaded NC (10 mM)	199 ± 15	0.06 ± 0.02	+40.0 ±4.5

NC: nanocapsules, NE: nanoemulsion.

**Fig 1:**
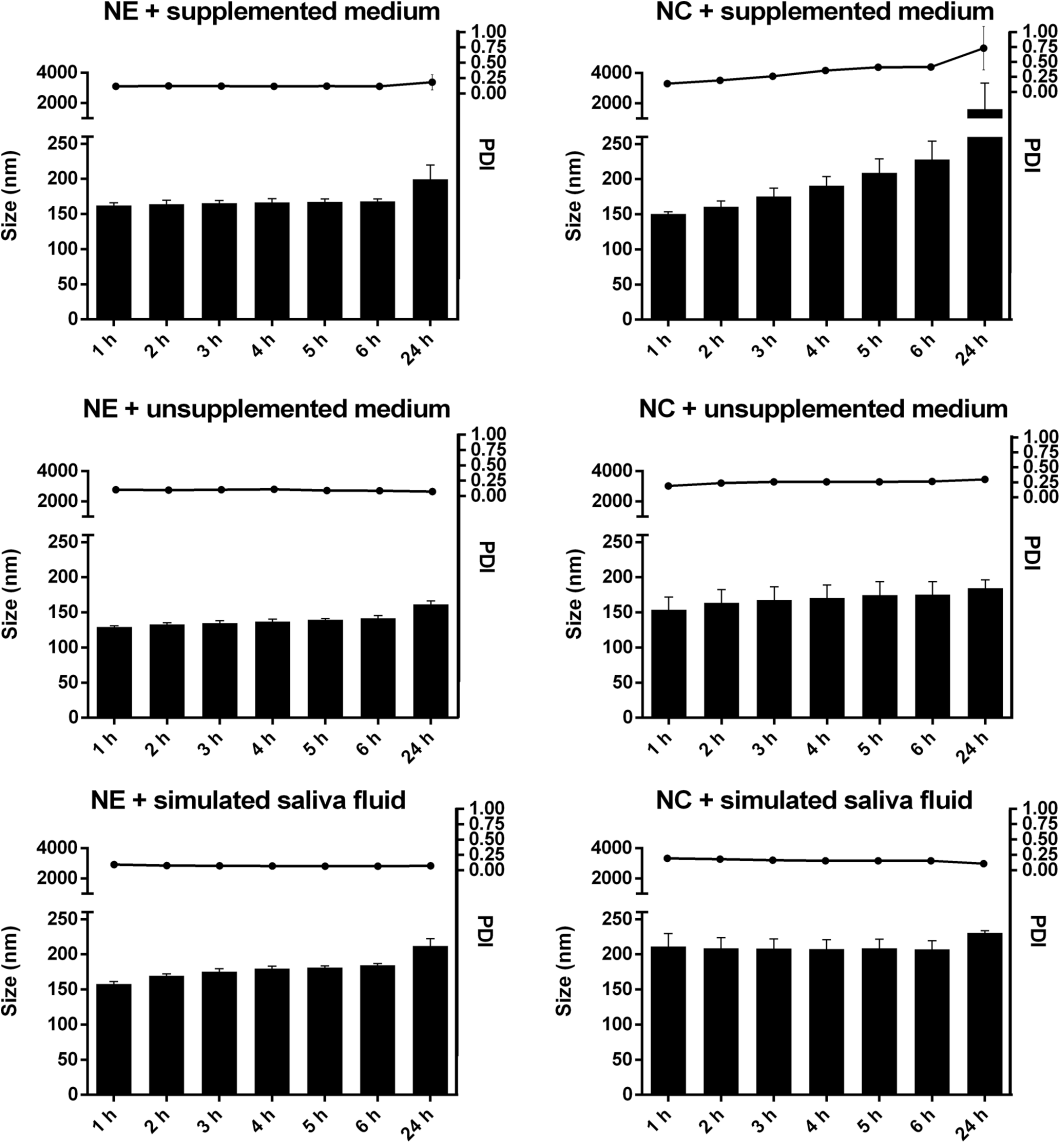
Stability measurements of nanoformulations in different physiological media. Evolution of diameter and polydispersity index (PDI) of chitosan-coated nanocapsules (NC) and nanoemulsions (NE) during incubation in different buffers at 37°C. Investigated media were Ham's F12 Nutrient Mixture with and without supplements and a simulated saliva fluid buffer containing α-amylase. Average sizes are represented as bars and the average polydispersity index (PDI) as dots connected by a line. Data are mean values ± SD (n = 3).

The stability in different media at 37°C was investigated by evaluating the evolution of the size distribution using DLS-NIBS. Cell culture medium of the type Ham's F12 Nutrient Mixture with and without supplements as well as a simulated saliva fluid buffer containing α-amylase were used. In Fig 1 the results of average size measurements together with the PDI over time in the different buffers are presented. After six hours the samples did not increase in average size or polydispersity. After 24 hours the size and PDI of the nanocapsules increased drastically when dispersed in supplemented cell culture medium. The results showed that the nanosystems were stable in various types of buffers. In case of the nanocapsules a presence of protein at higher concentrations seemed to destabilize the formulation as we observed precipitation after 24 hours when exposed to supplemented medium containing 10% of fetal calf serum. The α-amylase of human saliva did not affect the stability of the nanosystems even though a hydrolytic activity of α-amylase has been reported for chitosan [34]. Either chitosan adsorbed at the shell of the nanocapsules is not available for the hydrolytic activity of the enzyme, or the chitosan used, bearing a fairly high DA (ca. 30%), is not a good substrate. Furthermore, the reaction conditions differ from those reported in this study.

**Fig 2:**
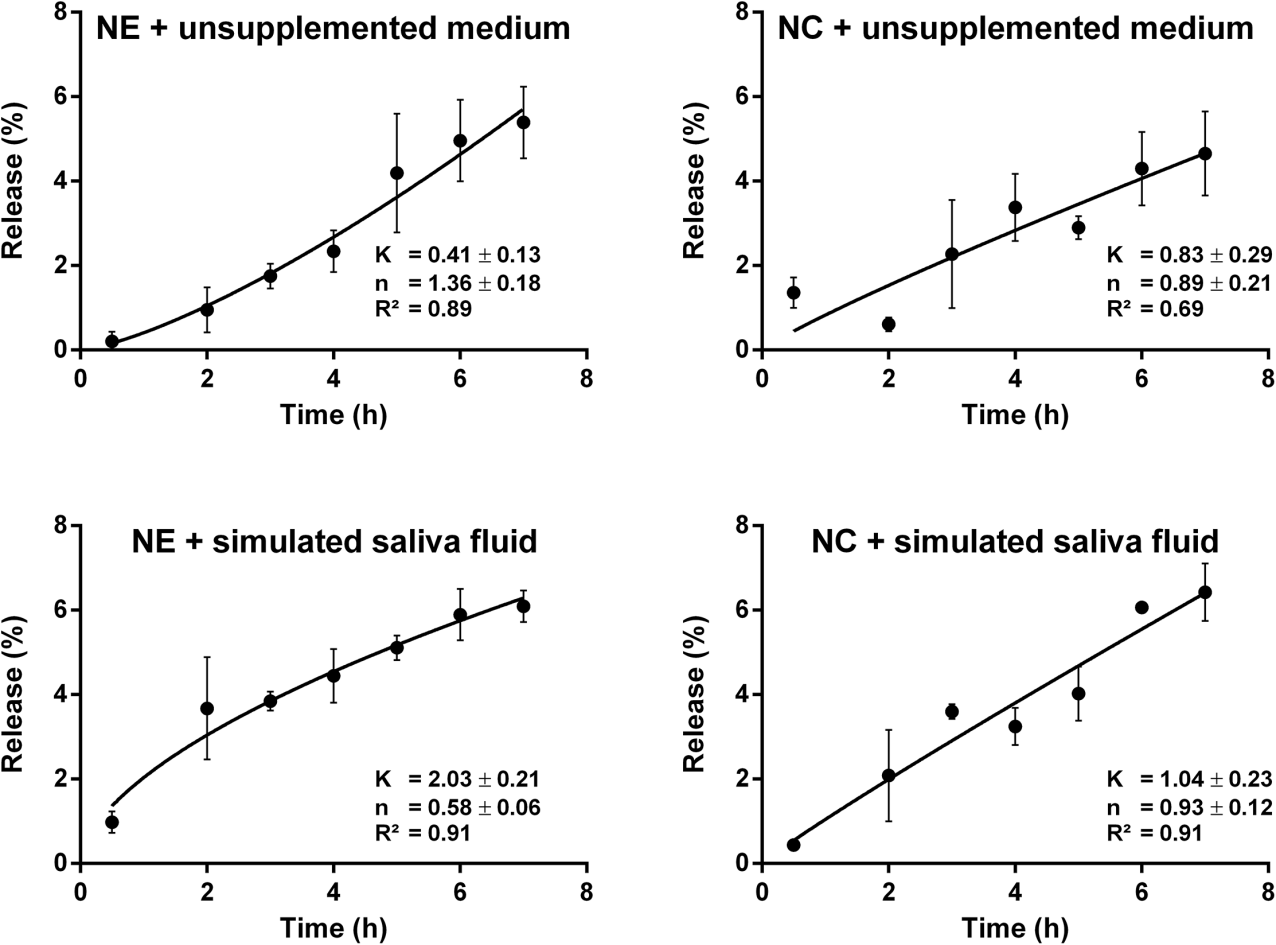
In vitro capsaicin release of nanoformulations. The capsaicin release of chitosan-coated nanocapsules (NC) and nanoemulsions (NE) into different buffers was carried out at 37°C. Investigated media were Ham's F12 Nutrient Mixture without supplements and a simulated saliva fluid. Non-linear fits were calculated according to the model of Korsmeyer and Peppas [25]. Data are mean values ± SD (n = 3).

For the cell culture medium without supplements and the simulated saliva fluid an *in vitro* release experiment at 37°C was performed with nanocapsules as well as nanoemulsions loaded with 10 mM of capsaicin. The released capsaicin amount over time is plotted in Fig 2. To characterize the release behavior of the formulations the model of Korsmeyer and Peppas [25] (see methods section) was applied (R^2^ = 0.69–0.91). The parameters of the fitted curves are shown in the diagrams. All of the formulations released about 6% of their load in the course of the experiment. Notice that the exponent *n* differed for the nanoemulsions between the different buffers (1.36 and 0.58) while it was comparable in case of the nanocapsules (0.89 and 0.93). This means that, according to the applied model, the nanoemulsion showed an anomalous diffusion behavior in saliva fluid while it released the compound with the kinetics of a super case-II transport in the medium. On the contrary, the chitosan coated formulation showed an almost ideal case-II transport behavior (n = 0.89) in both buffers. This indicates that the presence of chitosan in the nanocapsule shell makes the formulation more resistant to the environmental influence of the buffer. In a previous study where MEM was used as buffer the chitosan coating also influenced the release behavior of the systems although the values for the parameter *n* differed from those reported in this study, nevertheless, the overall released amounts of capsaicin were also comparable [12].

### Cytotoxicity experiments

**Fig 3:**
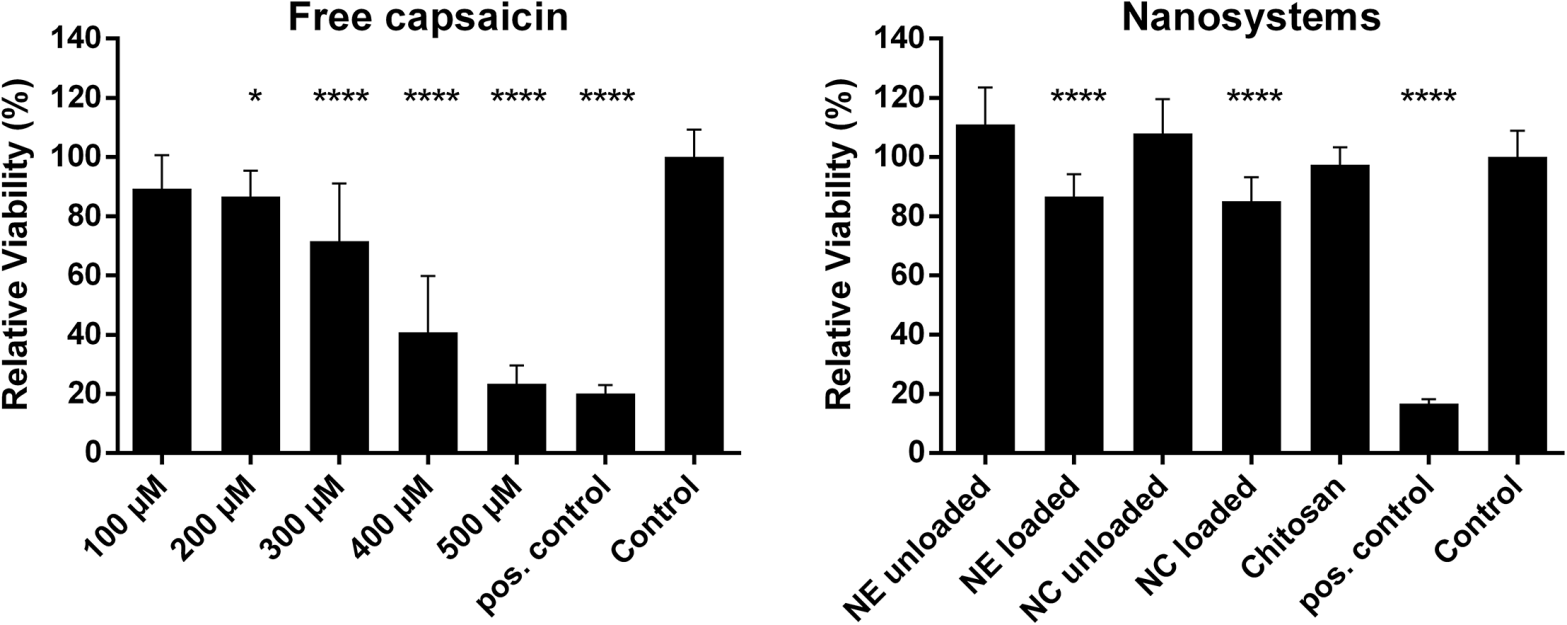
Cytotoxicity of different formulations against TR146 cells determined using the MTT assay. Relative cell viability following treatment with free capsaicin at increasing concentrations (Left). Relative cell viability following treatment with the different nanoformulations or their constituents at a universal concentration of 500 μM capsaicin (Right). For all experiments, cells were incubated for 3 h. Mean values ± SD. (n = 3, * p < 0.05, **** p < 0.0001). NC = nanocapsules; NE = nanoemulsion.

We carried out cytotoxicity experiments to see how the encapsulation changes the interaction between capsaicin and the cells using the MTT assay. For these experiments we used the buccal cell line TR146 as a model of human buccal epithelium. An incubation time of 3 hours was chosen. Fig 3 shows the cytotoxic effect of capsaicin in the free form with increasing concentrations and encapsulated in the two nanoformulations at a concentration of 500 μM. The same amounts of unloaded formulations and constituents were investigated as controls. A dose dependent reduction in metabolic competence was observable when the free compound was added to cells. A concentration of 200 μM was sufficient to induce a statistically significant reduction (p < 0.05) in comparison to the control. More concentrated samples showed an even higher level of significance (p < 0.0001). The unloaded nanosystems and constituents left the cells unaffected. The loaded nanoformulations significantly reduced the cell viability (p < 0.0001) to ~ 80% which is less pronounced than capsaicin in its free form (~ 20%) at the same concentration (500 μM). This confirmed the result of the release experiments that the formulations did not release capsaicin immediately which would have led to a much higher cytotoxic effect. These results concurred with those of a previous studies conducted with epithelial MDCK-C7 [12] and Caco-2 cells (Unpublished data).

### Sensory determination of nanoformulation thresholds

**Fig 4:**
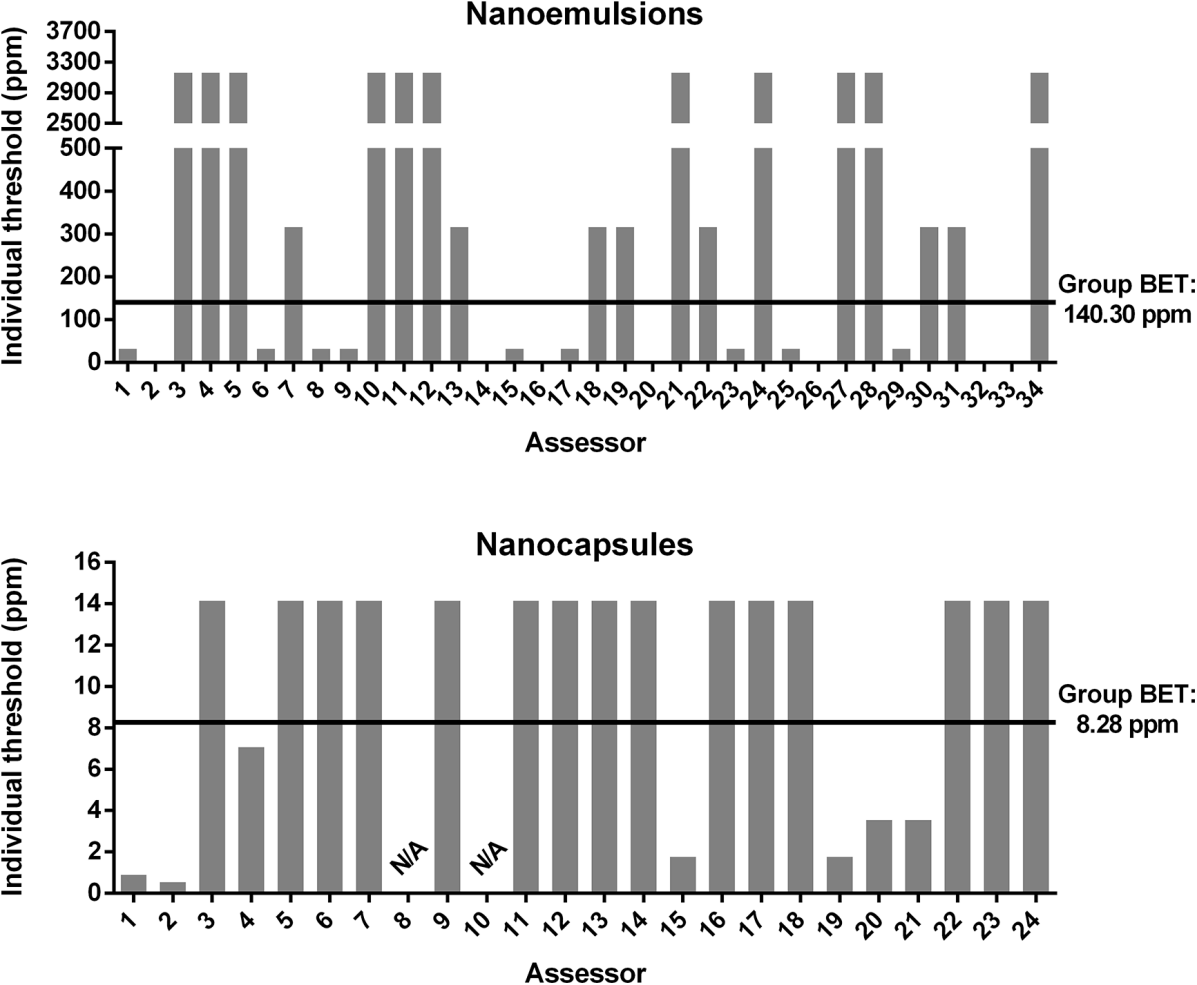
Individual best estimate threshold (BET) of unloaded nanoformulations. Individual BET of each panelist in comparison to the group BET for the unloaded nanoemulsion and nanocapsules.

For illustration purposes the individual BET values of the panelists in comparison to the group’s BET for the unloaded nanoformulations are shown in Fig 4. The experiments of unloaded nanoemulsions offered a group BET of 140.30 (SD log_10_ ± 1.15) ppm. Here 52.94% of the individual BETs were higher than the group BET and 47.06% were lower. Group BET of unloaded nanocapsules yielded 8.28 (SD log_10_ ± 0.38) ppm. 62.50% of the individual BETs showed higher results and 29.17% are lower than the group BET. These results confirmed the surmise made after the pretests that nanoemulsions could only be detected at higher concentrations in comparison to unloaded nanocapsules suggesting that a chitosan coating has an influence on the sensory perception of the formulation. Earlier studies using chitosan as a coating for cut fruits reported that the flavor of the fruits was not influenced [35]. By contrast, other studies have evaluated the sensory perception of astringency on chitosan solutions [36]. The minimum perceived astringent concentration of chitosan in solution ranged between 0.025 and 0.05%. This values are about 30 to 60-fold greater than those detected by our sensory panel. The differences in the detection thresholds of chitosan between our panel and that of previous studies can stem either on the state of chitosan (i.e. as a polymer solution or as a thin coat of emulsion droplets) as well as on the type of sensory test applied. The standard deviation of the BETs of unloaded emulsions was higher because of greater intervals between the single proffered concentrations. According to DIN standards the reference substances for threshold detections for the basic tastes are caffeine and citric acid with a concentration of 200 ppm, sodium chloride with 1300 ppm and sucrose with 6000 ppm [28]. Besides, the threshold concentration of capsaicin in water as an example for a trigeminal sensation is 0.08 ppm [37]. So, BET thresholds of unloaded formulations were comparable to gustatory and trigeminal sensation thresholds. This made them suitable for oral applications in the field of pharmaceuticals or food. Moreover, account must be taken of the fact that also the tactile sensation could have had an influence.

### Sensory determination of capsaicin thresholds in nanoformulations

**Fig 5:**
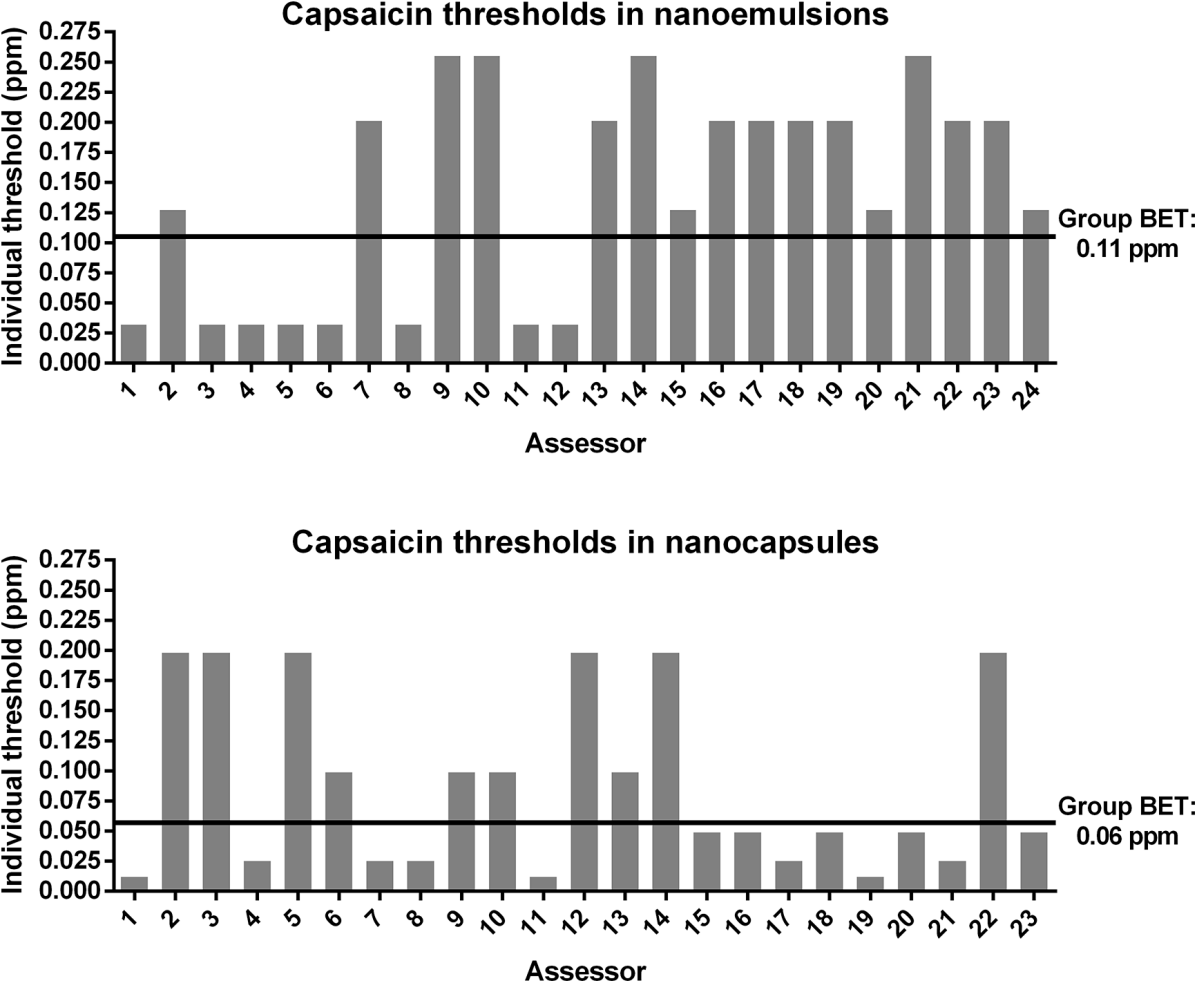
Individual best estimate threshold (BET) of capsaicin-loaded nanoformulations. Individual BET of each panelist in comparison to the group BET for the capsaicin-loaded nanoemulsion and nanocapsules.

The group’s best estimate threshold (BET) of capsaicin in nanocapsules yielded 0.06 (SD log_10_ ± 0.43) ppm while capsaicin in nanoemulsion showed a BET value of 0.11 (SD log_10_ ± 0.38) ppm (Fig 5). Both thresholds differed significantly in a one-way variance analysis (*p* < 0.05). Correspondingly, the real BET of capsaicin in the nanocapsules might be in the range of 0.04 to 0.07 ppm and in nanoemulsion in the range of 0.08 to 0.14 ppm.

**Fig 6:**
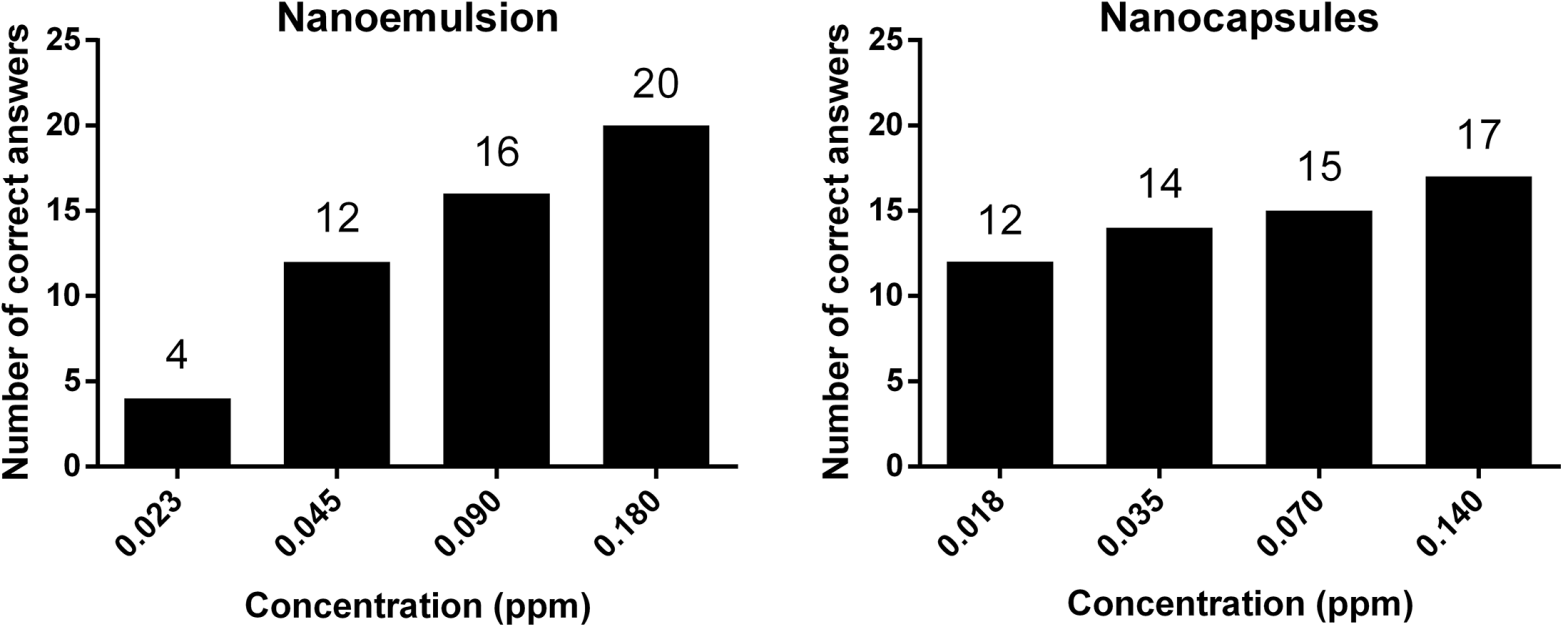
Number of correct answers during best estimate threshold (BET) study. Number of correct answers according to different concentrations of capsaicin for the nanoemulsion and the nanocapsules.

The number of correct answers for various dilutions of the capsaicin loaded nanoemulsion and nanocapsules is shown in Fig 6. Notice that this value increased with each ascending dilution step. It was observable that the first dilution step of the nanoemulsion (Fig 4) showed four correct answers much less than the following one (discrepancy of eight correct responses). Differences between the other dilution steps were not that high.

For the loaded nanocapsules 43.5% of the individual BETs were lower than the group BET of 0.06 ppm. For the loaded nanoemulsion 33.3% of the individual BETs were lower than the group BET of 0.11 ppm. The results of different threshold studies for capsaicin are shown in Table 3. As expected, the number of correct answers increased with an ascending capsaicin concentration. This indicated that the panel was trained effectively and the results were reproducible. But it is unclear why there was a high discrepancy between the concentrations of 0.02 and 0.05 ppm of the nanoemulsion with eight correct answers (Fig 6). A possible interpretation is that the panelist guessed which one was the odd sample at these low concentrations and most of them guessed wrongly because the concentration is below the threshold. In comparison to the BETs of other studies (pure capsaicin in aqueous solutions) the encapsulated formulations did not show a higher threshold, contrary to our expectations. In previous studies conducted by our own groups we calculated a BET of 0.09 ppm for pure capsaicin in water [38]. That means a higher rating than the range of the nanocapsules but similar to the range of the nanoemulsion with a minimum threshold of 0.08 ppm. In a following study we estimated a BET of 0.08 ppm and in consideration of the standard deviation it also matched with the range of the nanoemulsion and even with the one of the nanocapsules in the present study [37]. In previous studies [39] a BET for unencapsulated capsaicin dissolved in ethanol lied within a range of 0.04 to 0.08 ppm. This value agrees with our results of the nanocapsules but not with the one of nanoemulsion which were rated higher. Furthermore in another study [40] capsaicin was dissolved in ethanol, too, and a BET within a range of 0.09 to 0.35 ppm was calculated. Compared to our results, the threshold of nanoemulsion corresponded with this BET of pure capsaicin but nanocapsules were rated lower. So our hypothesis that encapsulation would result in an increasing of the threshold of capsaicin could not finally be confirmed. As also shown in Table 4, nanoformulations were rated equally as unencapsulated capsaicin or even lower. A possible explanation for this phenomenon could be a rapid hydrolysis of the nanoformulations as soon as being administered. We also think that direct contact with the surface of the oral cavity can lead to interactions between the nanoformulations and epithelial cells. In case of the nanoemulsion, the lipid bilayer of the cell membrane may interact with lecithin. This could lead to an immediate fusion of the emulsions with the cell membrane thus a discharge of the cargo could be suspected. In case of the chitosan-coated nanocapsules a similar effect might be envisaged though the interaction between positively-charged residues on the chitosan polymer with the negatively-charged glycocalyx on the cell surface may also play a role [41]. Further studies are required to investigate these phenomena in greater depth.

**Table 3:** Comparison of the thresholds of free capsaicin in literature with the present study.

Author	Threshold (ppm)	Emulsifier
Krajewska and Powers (1988)	0.04–0.08	Ethanol
Schneider et al. (2011)	0.09	Polysorbate 80
Schneider et al. (2014a)	0.08	Polysorbate 80
Sizer and Harris (1985)	0.09–0.35	Ethanol
This study	0.04–0.07 (NC)	Polysorbate 80
	0.08–0.14 (NE)	

NC: nanocapsules, NE: nanoemulsion.

**Table 4:** Mean pungency ratings of the three kinds of formulations.

Capsaicin concentration	Type of encapsulation	Sensory Rating (I_max_)	Standard deviation	Classification ^a^
2 ppm	C	4.20	1.53	a
2 ppm	NC	2.87	1.22	b
2 ppm	NE	3.69	1.23	c
6 ppm	C	7.28	1.28	d
6 ppm	NC	5.47	1.13	e
6 ppm	NE	4.91	1.51	f

C: unencapsulated; NC: nanocapsules, NE: nanoemulsion.

^a^ a, b, c, d, e, f–*I*_max_ mean values with different letters in columns are significantly different

*p* < 0.05.

### Sensory evaluation of pungency in various forms of encapsulation

**Fig 7:**
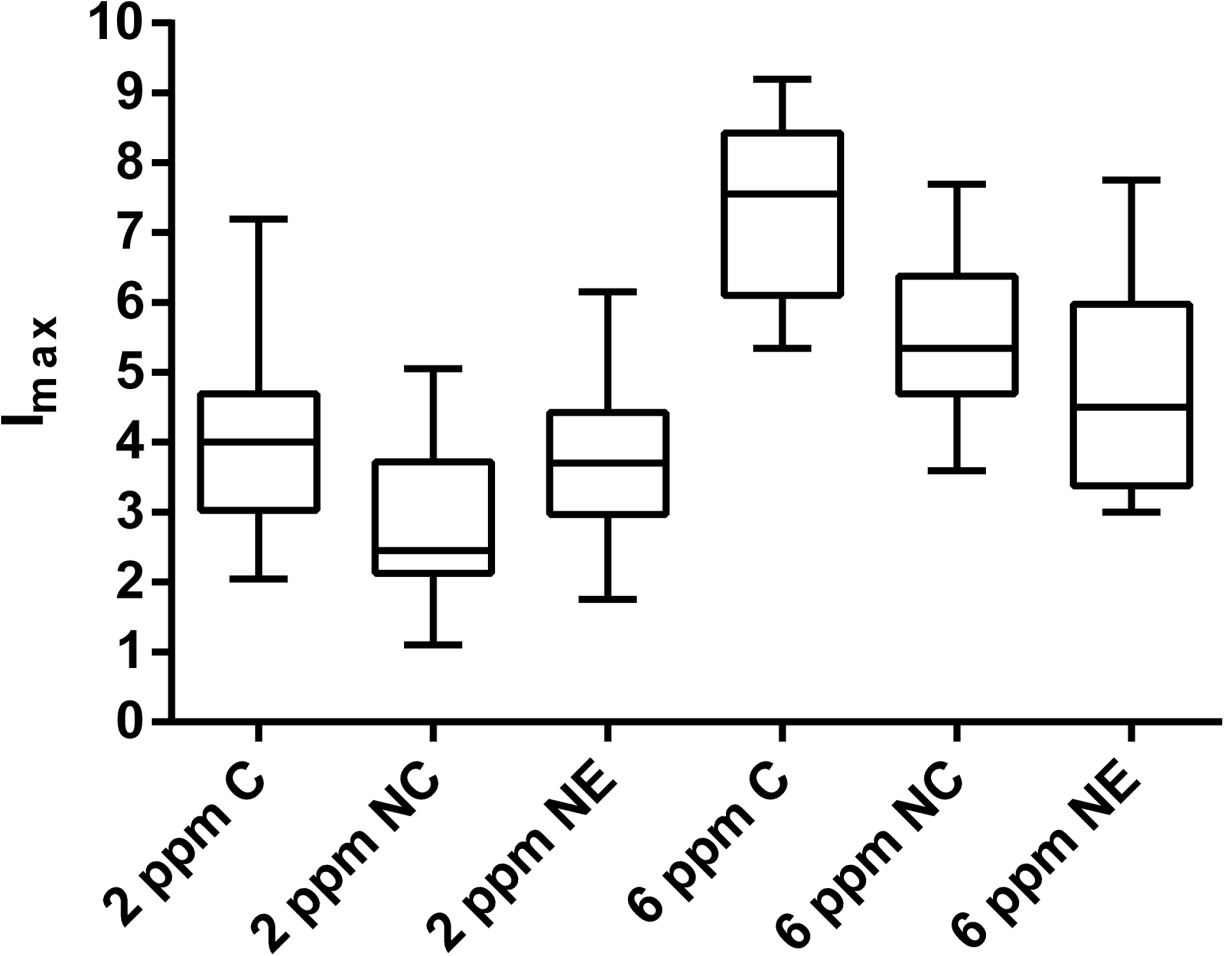
Pungency intensity tests of the nanoformulations in comparison to free capsaicin. Sensory evaluation of pungency of the different nanoformulations in comparison to the free compound at different concentrations (scale: 0–10; 0 = “not detectable”, 10 = “extremely pungent”). C = free capsaicin; NC = nanocapsules; NE = nanoemulsion. For statistical analysis refer to Table 4.

The results of the time intensity test are shown in Table 4. Based on the individual data of double identification, the mean values of *I*_max_ were calculated. The three-way ANOVA (panelist, kind of formulation and replicates) established that all samples varied from each other significantly both in different concentrations and different formulations. The present results showed a trend that the unencapsulated capsaicin was assessed higher in pungency than the encapsulated formulations with the same concentrations. So in the case of 2 ppm, unencapsulated capsaicin was rated 31.7% higher than the capsules and 12.1% higher than the emulsion. Furthermore 6 ppm unencapsulated capsaicin was rated 24.9% higher than the capsules and 32.6% higher than the emulsion with the same concentration. As a reference, unencapsulated capsaicin differed between 2 and 6 ppm significantly and 6 ppm was rated much higher (42.3%) than 2 ppm. There is no clear trend whether capsules or emulsion were judged more pungent because 2 ppm emulsion was rated 22.2% higher than capsules with the same concentration while 6 ppm capsules were rated 10.2% higher than 6 ppm emulsion. For better illustration of the following parameters the data were transformed into box plots (Fig 7). These box plots show the median, the lowest and highest value and the distribution of the data values.

As shown in Fig 7 encapsulation reduced perception of pungency significantly, as we had expected. A possible reason for this could be the complexity of the matrix of both formulations. We previously investigated the influence of the food matrix to the perception of pungency [30]. It was stated that the more complex the matrix was the less pungency of capsaicin was perceived. Depending on this the formulations consisted of many ingredients similar to the complex food matrices in this study. Contrary to the results of time intensity measurement the results of the threshold experiments in the present study showed that there was no proven difference between formulations and free capsaicin. This could be explained by the fact that the formulations could have a better masking effect in higher concentrations. For validation further studies should be performed with concentrations higher than 6 ppm. Pungency ratings of both formulations differed significantly, too. There was no clear trend that the nanoemulsion showed a better masking effect on the perception of pungency although the threshold of the nanoemulsion was rated higher than the one of the nanocapsules. The relative high statistical spread in Fig 7 could be explained by the individual difference in pungency perception of each single panelist. The results showed consistence to other studies analyzing the sensory masking efficiency of encapsulated systems. In another study [42] the efficiency of the microencapsulation of sildenafil citrate for masking bitter taste was analyzed. It was stated that the bitterness score decreased with increasing polymer ratios using encapsulation technology. This was consistent with our results that encapsulation reduced the sensation of pungency. Similar results by investigating the possibility of adding microencapsulated fish oil as an ingredient to bread products were described [43]. This study showed that encapsulation technology was able to mask the fish taste.

## Conclusion

In this study we showed that the investigated nanoformulations loaded with capsaicin were stable in different types of physiological buffers and that encapsulation of capsaicin within these systems allows a slow controlled release. The release behavior in different buffers was influenced by the chitosan coating. Both of the formulations reduced the cytotoxicity of the compound *in vitro* (TR146 cells). In the sensory study the 3-AFC-test established a threshold for the unloaded formulations of 140.30 (SD log_10_ ± 1.15) ppm for the nanoemulsions and 8.28 (SD log_10_ ± 0.38) ppm for the nanocapsules showing that the use of chitosan decreased the threshold of the formulation which suggests that a chitosan coating has an impact of taste perception. For encapsulated capsaicin thresholds of 0.06 (SD log_10_ ± 0.43) ppm for the nanocapsules and 0.11 (SD log_10_ ± 0.38) ppm for the nanoemulsion was detrmined. This is in the range of unencapsulated capsaicin. The pungency of free capsaicin was rated more intense than for its encapsulated counterparts at identical concentrations (2 and 6 ppm). Statistical analysis of the results showed that there was a significant difference between encapsulated and unencapsulated formulations. This confirms the hypothesis that encapsulation does control the release of capsaicin. There was, however, no trend whether capsules or emulsions generally had a better masking effect.

## References

[1] Korel F, Bagdatlioglu N, Balaban MÖ, Hisil Y. Ground red peppers: Capsaicinoids content, scoville scores, and discrimination by an electronic nose. J Agric Food Chem. 2002;50: 3257–3261. 12009995 10.1021/jf010537b

[2] Julius D, Basbaum AI. Molecular mechanisms of nociception. Nature. 2001;413: 203–210. 11557989 10.1038/35093019

[3] Govindarajan VS, Sathyanarayana MN. Capsicum—production, technology, chemistry, and quality. Part V. Impact on physiology, pharmacology, nutrition, and metabolism; structure, pungency, pain, and desensitization sequences. Crit Rev Food Sci Nutr. 1991;29: 435–474. 2039598 10.1080/10408399109527536

[4] Meghvansi MK, Siddiqui S, Khan MH, Gupta VK, Vairale MG, Gogoi HK, et al Naga chilli: A potential source of capsaicinoids with broad-spectrum ethnopharmacological applications. J Ethnopharmacol. 2010;132: 1–14. 10.1016/j.jep.2010.08.034 20728519

[5] Nascimento PLA, Nascimento TCES, Ramos NSM, Silva GR, Gomes JEG, Falcão REA, et al Quantification, Antioxidant and Antimicrobial Activity of Phenolics Isolated from Different Extracts of Capsicum frutescens (Pimenta Malagueta). Molecules. 2014;19: 5434–5447. 10.3390/molecules19045434 24879587 PMC6271728

[6] Kurita S, Kitagawa E, Kim C-, Momose Y, Iwahashi H. Studies on the antimicrobial mechanisms of capsaicin using yeast DNA microarray. Bioscience, Biotechnology and Biochemistry. 2002;66: 532–536. 10.1271/bbb.66.532 12005045

[7] Zhang LL, Liu DY, Ma LQ, Luo ZD, Cao TB, Zhong J, et al Activation of transient receptor potential vanilloid type-1 channel prevents adipogenesis and obesity. Circ Res. 2007;100: 1063–1070. 17347480 10.1161/01.RES.0000262653.84850.8b

[8] Shiobara T, Usui T, Han J, Isoda H, Nagumo Y. The reversible increase in tight junction permeability induced by capsaicin is mediated via cofilin-actin cytoskeletal dynamics and decreased level of occludin. PLoS ONE. 2013;8. 10.1371/journal.pone.0079954 PMC3832373 24260326

[9] Tsukura Y, Mori M, Hirotani Y, Ikeda K, Amano F, Kato R, et al Effects of capsaicin on cellular damage and monolayer permeability in human intestinal Caco-2 cells. Biological and Pharmaceutical Bulletin. 2007;30: 1982–1986. 17917278 10.1248/bpb.30.1982

[10] Turgut C, Newby B-, Cutright TJ. Determination of Optimal Water Solubility of Capsaicin for its Usage as a Non-toxic Antifoulant. Environmental Science and Pollution Research. 2004;11: 7–10. 15005134 10.1065/espr2003.12.180

[11] Goycoolea FM, Valle-Gallego A, Stefani R, Menchicchi B, David L, Rochas C, et al Chitosan-based nanocapsules: Physical characterization, stability in biological media and capsaicin encapsulation. Colloid Polym Sci. 2012;290: 1423–1434.

[12] Kaiser M, Pereira S, Pohl L, Ketelhut S, Kemper B, Gorzelanny C, et al Chitosan encapsulation modulates the effect of capsaicin on the tight junctions of MDCK cells. Sci Rep. 2015;5. 10.1038/srep10048 PMC4429556 25970096

[13] Menchicchi B, Fuenzalida JP, Bobbili KB, Hensel A, Swamy MJ, Goycoolea FM. Structure of chitosan determines its interactions with mucin. Biomacromolecules. 2014;15: 3550–3558. 10.1021/bm5007954 25122160

[14] Yeh T, Hsu L, Tseng MT, Lee P, Sonjae K, Ho Y, et al Mechanism and consequence of chitosan-mediated reversible epithelial tight junction opening. Biomaterials. 2011;32: 6164–6173. 10.1016/j.biomaterials.2011.03.056 21641031

[15] Xing F, Cheng G, Yang B, Ma L. Microencapsulation of capsaicin by the complex coacervation of gelatin, acacia and tannins. J Appl Polym Sci. 2004;91: 2669–2675.

[16] Xing F, Cheng G, Yi K. Study on the antimicrobial activities of the capsaicin microcapsules. J Appl Polym Sci. 2006;102: 1318–1321.

[17] Choi A-, Kim C-, Cho Y-, Hwang J-, Kim C-. Characterization of Capsaicin-Loaded Nanoemulsions Stabilized with Alginate and Chitosan by Self-assembly. Food and Bioprocess Technology. 2011;4: 1119–1126.

[18] Jincheng W, Sihao C. Preparation and characterization of microcapsules containing capsaicin. J Appl Polym Sci. 2010;116: 2234–2241.

[19] Zhu Y, Peng W, Zhang J, Wang M, Firempong CK, Feng C, et al Enhanced oral bioavailability of capsaicin in mixed polymeric micelles: Preparation, in vitro and in vivo evaluation. Journal of Functional Foods. 2014;8: 358–366.

[20] Choi AY, Kim C-, Park HY, Kim HO, Lee NR, Lee KE, et al Pharmacokinetic characteristics of capsaicin-loaded nanoemulsions fabricated with alginate and chitosan. J Agric Food Chem. 2013;61: 2096–2102. 10.1021/jf3052708 23414078

[21] Kim JH, Ko JA, Kim JT, Cha DS, Cho JH, Park HJ, et al Preparation of a capsaicin-loaded nanoemulsion for improving skin penetration. J Agric Food Chem. 2014;62: 725–732. 10.1021/jf404220n 24417234

[22] Rinaudo M, Milas M, Le Dung P. Characterization of chitosan. Influence of ionic strength and degree of acetylation on chain expansion. Int J Biol Macromol. 1993;15: 281–285. 8251442 10.1016/0141-8130(93)90027-j

[23] Peh KK, Wong CF. Polymeric films as vehicle for buccal delivery: swelling, mechanical, and bioadhesive properties. Journal of pharmacy & pharmaceutical sciences: a publication of the Canadian Society for Pharmaceutical Sciences, Société canadienne des sciences pharmaceutiques. 1999;2: 53–61. 10952770

[24] Arhakis A, Karagiannis V, Kalfas S. Salivary alpha-amylase activity and salivary flow rate in young adults. Open Dentistry Journal. 2013;7: 7–15. 10.2174/1874210601307010007 23524385 PMC3601341

[25] Korsmeyer RW, Gurny R, Doelker E, Buri P, Peppas NA. Mechanisms of solute release from porous hydrophilic polymers. Int J Pharm. 1983;15: 25–35. 10.1002/jps.2600721021 6644570

[26] Costa P, Sousa Lobo JM. Modeling and comparison of dissolution profiles. European Journal of Pharmaceutical Sciences. 2001;13: 123–133. 11297896 10.1016/s0928-0987(01)00095-1

[27] Denizot F, Lang R. Rapid colorimetric assay for cell growth and survival—Modifications to the tetrazolium dye procedure giving improved sensitivity and reliability. J Immunol Methods. 1986;89: 271–277. 3486233 10.1016/0022-1759(86)90368-6

[28] DIN EN ISO 8586:2014–05. Sensory analysis—General guidelines for the selection, training and monitoring of selected assessors and expert sensory assessors (ISO 8586:2012); German version EN ISO 8586:2014.

[29] DIN 10962:1997–10. Areas for sensory analysis—Specifications of testrooms.

[30] Schneider DJ, Seuß-Baum I, Schlich E. Relationship between pungency and food components—A comparison of chemical and sensory evaluations. Food Quality and Preference. 2014;38: 98–106.

[31] ISO 13301:2002–09. Sensory analysis—Methodology—General guidance for measuring odour, flavour and taste detection thresholds by a three-alternative forced-choice (3-AFC) procedure.

[32] DIN 10970:2002–09. Sensory analysis—Time intensity test.

[33] Calvo P, Vila-Jato JL, Alonso MJ. Evaluation of cationic polymer-coated nanocapsules as ocular drug carriers. Int J Pharm. 1997;153: 41–50.

[34] Wu S. Preparation of water soluble chitosan by hydrolysis with commercial a-amylase containing chitosanase activity. Food Chem. 2011;128: 769–772.

[35] Chien P-, Sheu F, Yang F-. Effects of edible chitosan coating on quality and shelf life of sliced mango fruit. J Food Eng. 2007;78: 225–229.

[36] Rodríguez MS, Albertengo LA, Vitale I, Agulló E. Relationship between astringency and chitosan-saliva solutions turbidity at different pH. J Food Sci. 2003;68: 665–667.

[37] Schneider DJ, Seuß-Baum I, Schlich E. Comparison between Chemical Senses Thresholds for Capsaicin and Dihydrocapsaicin in Aqueous Solutions and Identification of the Area of Burning Sensation. Advance Journal of Food Science and Technology. 2014;6: 36–41.

[38] Schneider DJ, Seuß-Baum I, Schlich E. Untersuchung zur Reizschwellenermittlung von Capsaicin unter Anwendung der 3-AFC Methode nach ISO 13301:2002, Poster: 6. DLG-Lebensmitteltage, Darmstadt. 2011.

[39] Krajewska AM, Powers JJ. Sensory Properties of Naturally Occurring Capsaicinoids. J Food Sci. 1988;53: 902–905.

[40] Sizer F, Harris N. The influence of common food additives and temperature on threshold perception of capsaicin. Chem Senses. 1985;10: 279–286.

[41] Kean T, Thanou M. Biodegradation, biodistribution and toxicity of chitosan. Adv Drug Deliv Rev. 2010;62: 3–11. 10.1016/j.addr.2009.09.004 19800377

[42] Yi E-, Kim J-, Rhee Y-, Kim S-, Lee H-, Park C-, et al Preparation of sildenafil citrate microcapsules and in vitro/in vivo evaluation of taste masking efficiency. Int J Pharm. 2014;466: 286–295. 10.1016/j.ijpharm.2014.03.001 24607218

[43] Davidov-Pardo G, Roccia P, Salgado D, León AE, Pedroza-Islas R. Utilization of different wall materials to microencapsulate fish oil evaluation of its behavior in bread products. American Journal of Food Technology. 2008;3: 384–393.

